# Improving child tuberculosis contact identification and screening in Lesotho: Results from a mixed-methods cluster-randomized implementation science study

**DOI:** 10.1371/journal.pone.0248516

**Published:** 2021-05-20

**Authors:** Yael Hirsch-Moverman, Andrea A. Howard, Joanne E. Mantell, Limakatso Lebelo, Koen Frederix, Aprielle Wills, Anneke C. Hesseling, Sharon Nachman, Llang B. Maama, Wafaa M. El-Sadr

**Affiliations:** 1 ICAP, Mailman School of Public Health, Columbia University, New York, New York, United States of America; 2 Department of Epidemiology, Mailman School of Public Health, Columbia University, New York, New York, United States of America; 3 Division of Gender, Sexuality and Health, at the New York State Psychiatric Institute and Department of Psychiatry, HIV Center for Clinical & Behavioral Studies, Columbia University Irving Medical Center, New York, New York, United States of America; 4 Department of Paediatrics and Child Health, Desmond Tutu TB Centre, Faculty of Medicine and Health Sciences, Stellenbosch University, Tygerberg, South Africa; 5 Pediatric Infectious Diseases, SUNY Stony Brook, Stony Brook, New York, United States of America; 6 Lesotho Ministry of Health National Tuberculosis Program, Maseru, Lesotho; UNITED STATES

## Abstract

**Background:**

Child tuberculosis (TB) contact management is recommended for preventing TB in children but its implementation is suboptimal in high TB/HIV-burden settings. The PREVENT Study was a mixed-methods, clustered-randomized implementation study that evaluated the effectiveness and acceptability of a community-based intervention (CBI) to improve child TB contact management in Lesotho, a high TB burden country.

**Methods:**

Ten health facilities were randomized to CBI or standard of care (SOC). CBI holistically addressed the complex provider-, patient-, and caregiver-related barriers to prevention of childhood TB. Routine TB program data were abstracted from TB registers and cards for all adult TB patients aged >18 years registered during the study period, and their child contacts. Primary outcome was yield (number) of child contacts identified and screened per adult TB patient. Generalized linear mixed models tested for differences between study arms. CBI acceptability was assessed via semi-structured in-depth interviews with a purposively selected sample of 20 healthcare providers and 28 caregivers. Qualitative data were used to explain and confirm quantitative results. We used thematic analysis to analyze the data.

**Results:**

From 01/2017-06/2018, 973 adult TB patients were recorded, 490 at CBI and 483 at SOC health facilities; 64% male, 68% HIV-positive. At CBI and SOC health facilities, 216 and 164 child contacts were identified, respectively (p = 0.16). Screening proportions (94% vs. 62%, p = 0.13) were similar; contact yield per TB case (0.40 vs. 0.20, p = 0.08) was higher at CBI than SOC health facilities, respectively. CBI was acceptable to caregivers and healthcare providers.

**Conclusion:**

Identification and screening for TB child contacts were similar across study arms but yield was marginally higher at CBI compared with SOC health facilities. CBI scale-up may enhance the ability to reach and engage child TB contacts, contributing to efforts to improve TB prevention among children.

## Introduction

The World Health Organization (WHO) estimates that 1.12 million children globally develop tuberculosis (TB) annually, with approximately 200,000 TB deaths despite the availability of effective TB prevention and treatment and universal vaccination at birth in high TB burden countries [1, 2]. Following exposure to Mycobacterium tuberculosis (M.tb), children who are young, malnourished or HIV-positive have a disproportionately high risk of developing disseminated TB or death [3, 4]. Progression to TB typically occurs in the first 12 months following M.tb infection, and the youngest children are at greatest risk; HIV-positive children are highly vulnerable to development of TB, regardless of antiretroviral treatment status and age [3]. Child TB contact management (CCM) is a highly efficient strategy to identify children with and at-risk for TB [5, 6]. The CCM cascade includes identifying and screening child contacts exposed to adults with TB and ensuring initiation and completion of TB preventive treatment (TPT) or TB treatment, as appropriate. However, a recent review of CCM implementation in high TB burden countries found substantial losses at each CCM cascade step [7]. Innovative strategies are needed to strengthen identification and management of child contacts in high TB burden areas.

Lesotho, a lower-middle income sub-Saharan African country with a population of 2.1 million [8], has one of the world’s highest TB incidence rates (611 per 100,000 [2]) and second highest HIV prevalence (25.6% [9]). Four percent of reported TB patients are children aged <15 years [2], low compared to similar settings [10, 11], most likely due to underdiagnosis and underreporting. In 2011, the Lesotho National TB Program adopted WHO’s CCM recommendations [12]. However, implementation has been limited, with no evidence-based strategies guiding practical CCM implementation [13, 14].

We conducted the PREVENT Study, a mixed-methods cluster-randomized implementation science study to evaluate the effectiveness and acceptability of a combination community-based intervention (CBI) versus standard of care (SOC) to identify child contacts of adult TB patients, screen them for TB, and provide eligible children with TPT. We report on CBI effectiveness and acceptability in identifying and screening TB-exposed household child contacts.

## Materials and methods

**Fig 1:**
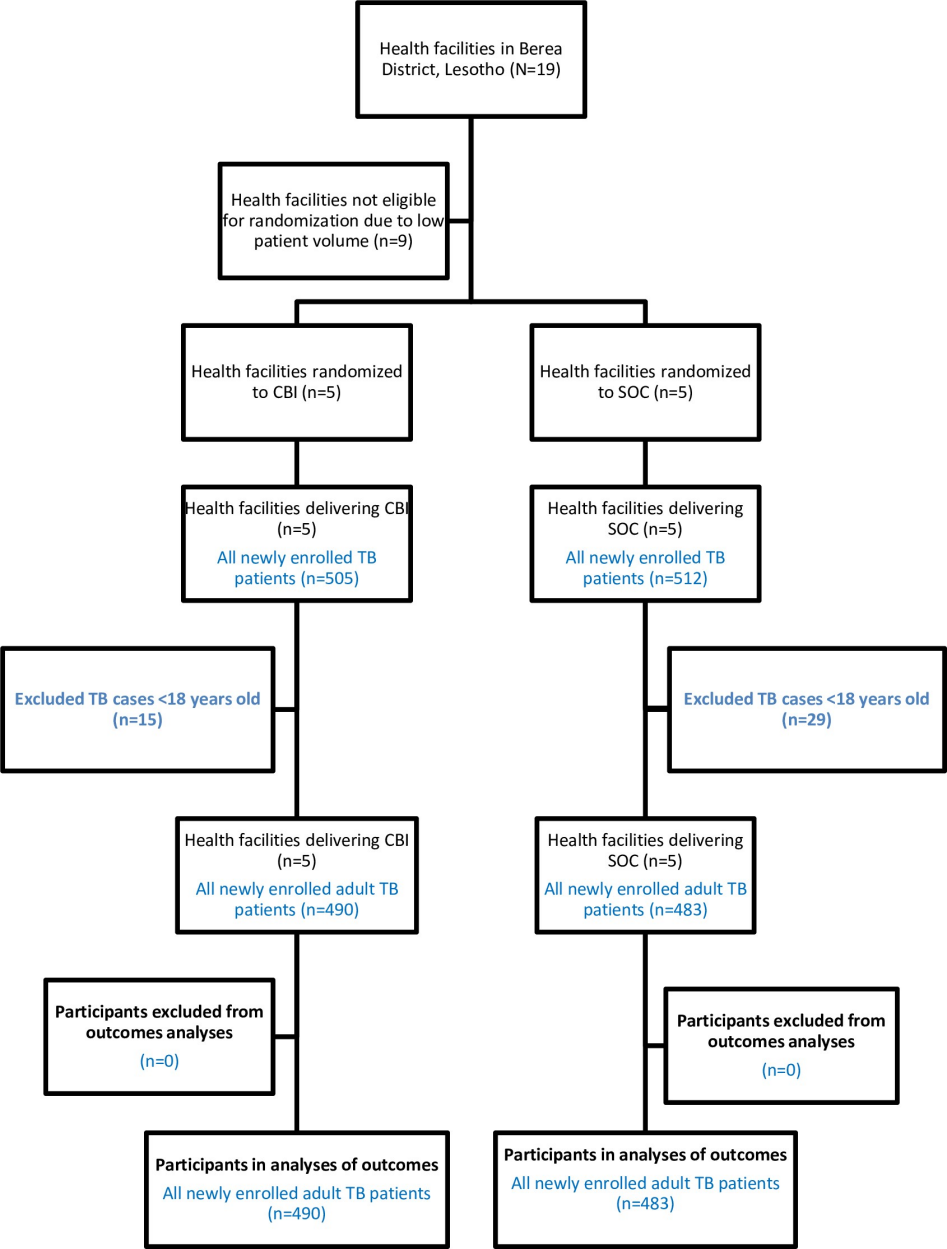
CONSORT diagram.

The PREVENT study protocol has been published elsewhere [15]. As this was a cluster-randomized trial, assignment to study arm was done at the health facility level and not at the individual participant level. Ten public health facilities (HFs; i.e., clusters) in Berea District, Lesotho, were randomized to deliver CBI or SOC, following stratification by facility type. The remaining nine health facilities were excluded from the sampling frame because of low TB patient case load (on average, <6 TB patients notified per quarter). Both hospitals (N = 2) and health centers (N = 8) were included to enhance generalizability of study findings as TB services are provided in both types of facilities in Lesotho (Fig 1). All patients at HFs assigned to the SOC arm received standard of care supported by the Lesotho MOH, whereas all patients at HFs assigned to the CBI arm received the standard of care plus the combination CBI. Healthcare providers (HCPs), patients, caregivers, and study staff were not blinded to the assigned study arm. CBI acceptability was assessed among healthcare providers and caregivers.

### Standard of care (SOC) intervention

At SOC-assigned HFs (SOC sites), usual care for CCM was delivered, including contact tracing, screening, and TPT provision. As per national guidelines, adult TB patients should be asked to bring child contacts to the HF for TB screening, where they are entered into a contact tracing register. Children with a positive symptom screen should be evaluated for TB; those with a positive sputum smear, GeneXpert, or radiograph should be treated for TB, and those with a negative screen or evaluation should be assessed for TPT eligibility. Village health workers (VHWs) are recommended to visit homes of TB patients and encourage child contacts to visit the HF for screening.

#### Community-based intervention (CBI)

At CBI-assigned HFs (CBI sites), the intervention was delivered to all adult TB patients and their child contacts and caregivers in addition to SOC. CBI was designed to holistically address the complex provider-, patient-, and caregiver-related barriers to prevention of childhood TB. It included the following components: (1) mentoring of nurses and facility-based lead VHWs (LVHWs) in CCM to enable them to inform adult TB patients and caregivers about the benefits of TPT; (2) visits by VHWs to all households of adult TB patients, and referral and accompaniment of all HIV-negative children <5 years and all HIV-positive children regardless of age to HFs; (3) intensive education on the importance of TB prevention, TPT provision and adherence by nurses and LVHWs to caregivers and children; (4) TPT adherence support via weekly calls and text messages and community-based VHW support, including follow-up with caregivers of children who missed appointments or reported nonadherence; and (5) monitoring and review of CCM data in quarterly multidisciplinary team meetings.

Quarterly refresher trainings and weekly mentorship were provided to nurses and LVHWs on-site by a study nurse mentor; LVHWs supervised and mentored VHWs. All nurses and LVHWs from CBI sites met as a team quarterly to review data and intervention activities, identify challenges, and develop solutions.

A Stakeholder Advisory Group (SAG), which included community representatives, was formed early and met regularly throughout the study to ensure community engagement.

#### Participants

All adult TB patients aged ≥18 years newly registered for TB treatment at the 10 participating HFs during the study period and their child contacts were included in the study. In addition, two groups of key informants from CBI sites were enrolled: (1) HCPs and (2) caregivers. HCP eligibility criteria were: aged ≥18 years, nurse or VHW working at or affiliated with CBI site, English- or Sesotho-speaking, and capacity for informed consent. Caregivers’ eligibility criteria were: aged ≥15 years, caregiver of a child contact in a CBI site, English- or Sesotho-speaking, and capacity for informed consent.

#### Data collection

All adult TB patients newly registered for TB treatment at participating HFs between January 2017 and June 2018 and their child contacts were included in the effectiveness analysis. Data were collected semiannually from TB registers, TB cards, and contact tracing registers using a standardized data abstraction tool. Acceptability of the intervention was assessed via semi-structured in-depth interviews with a purposively selected sample of HCPs (small group) and caregivers (individual). We used a sequential explanatory design phase [16], where quantitative data collection and analysis were followed by collection and analysis of qualitative data in an effort to explain our quantitative results and explore CBI acceptability and utilization of intervention components. This comprehensive approach allows for a greater understanding of how and why particular experiences and social realities of healthcare providers, children, and caregivers influence their attitudes and perceptions about TB prevention in child contacts. Questions relevant to this analysis addressed identification and TB screening of child contacts. Interviews were audio-recorded, transcribed verbatim, translated, anonymized and subjected to textual analysis.

#### Outcomes

The primary outcome for this analysis was yield of child contacts, defined as number of child contacts identified and screened per adult TB patient diagnosed during the study period. Secondary outcomes included: (1) number of child contacts identified per adult TB patient diagnosed; (2) proportion of child contacts screened among those identified; and (3) CBI acceptability defined as the perception among key stakeholders that CBI was satisfactory for identification and screening of child contacts.

#### Design assumptions and data analysis

*Quantitative*. We estimated that CBI will increase number of identified child contacts per adult TB patient from an average of 0.5 (based on the Lesotho National TB Program estimates from 2011) to 2.0 for each adult TB patient. In 2011, 1,132 new TB patients were diagnosed in 21 HFs in Berea district, for an average of 54 new patients per HF. Based on these assumptions and available data on number of TB cases, we calculated that a sample size of 5 clusters per study arm with 75 children per cluster would achieve 91% power to detect a difference of 1.5 between group means (0.5 child contacts per adult TB patient in SOC and 2.0 in CBI) using a two-sided t-test with significance level of 0.05 and a standard deviation of 2.5 (PASS 2008, NCSS Statistic Software).

An intent-to-treat analysis was used for the effectiveness analyses. Generalized linear mixed models were applied to test for differences in outcome between study arms. Models included fixed effects for study arm and random effects for study site to adjust for potential non-independence of observations.

*Qualitative*. We used thematic analyses [17] as the framework for data inquiry and CBI acceptability analysis, which helped to reveal the complex, social pathways that impact TB prevention efforts among child contacts. We used an iterative analytic process to facilitate a comprehensive understanding of participants’ perspectives. Two investigators independently reviewed five transcripts to develop a preliminary coding scheme. We used a “negotiated agreement approach” to ensure consistent interpretation and application of codes, which increases coding reliability [18, 19]. Once consensus on codes was achieved, the final coding scheme was applied to the full set of transcripts using Dedoose, a qualitative software program for systematic data management and analysis [20]. Typical quotations are used to illustrate the themes.

#### Ethics

The protocol was approved by the Columbia University Irving Medical Center Institutional Review Board (Ref AAAN7358) and the Lesotho National Health Research and Ethics Committee (Ref ID78-2015). Both entities deemed the medical record review as eligible for waiver of individual consent. In-depth interview participants provided written and informed consent. The study was registered at ClinicalTrials.gov (NCT02662829).

## Results

### CBI effectiveness

**Fig 2:**
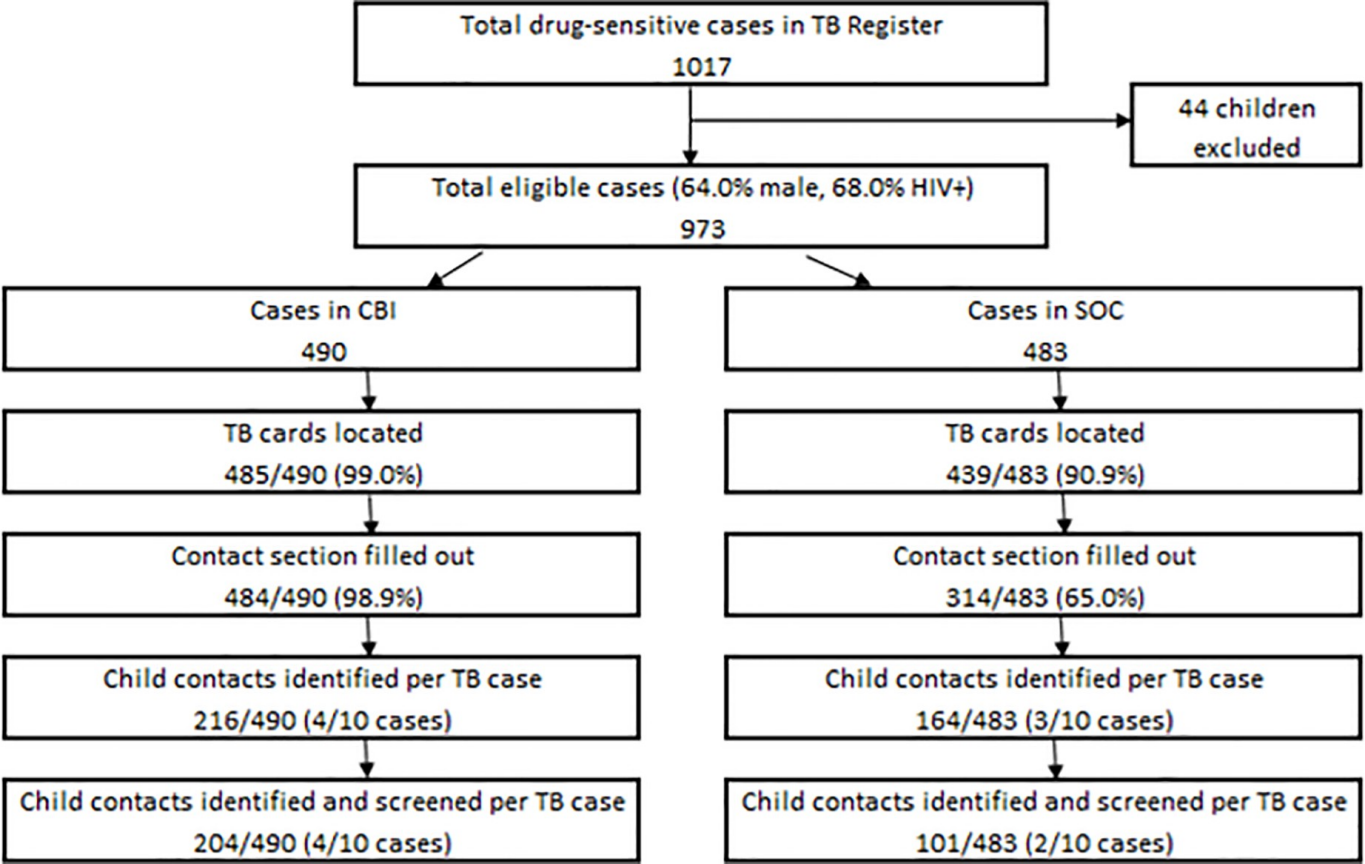
Study flow.

During the study period, 1,017 new TB patients were registered at the 10 study sites, including 973 adults and 44 children. Among adult TB patients, mean age was 44±15 years, 64.0% were male, and 68.0% were HIV-positive, with no differences between study arms. Of 973 adult TB cases, 490 were recorded at CBI and 483 at SOC sites (Fig 2). At CBI sites, TB cards were located for 99.0% (485/490) of TB patients and 98.8% (484/490) had a completed contact section compared with SOC sites, where 90.9% (439/483) of TB cards were located and 65.0% (314/483) had a completed contact section. At CBI sites, 216 child contacts were reported by 490 adult TB patients and at SOC sites 164 child contacts were identified by 483 adult TB patients (0.44 vs. 0.34 child contacts identified per adult TB patient, respectively, p = 0.13).

Almost all child contacts identified on adult TB cards at CBI sites were screened for TB compared with less than two-thirds at SOC sites (94.4% vs. 61.6%, p = 0.13). Among child contacts screened, 16.7% were aged <1, 29.0% aged 1–2, and 52.8% aged 3-<5 years, 51.6% were male, and 2.6% were HIV-exposed or HIV-positive, with no differences between study arms. The yield of child contacts who were identified and screened per adult TB patient was marginally higher at CBI than SOC sites (0.40 vs. 0.20, p = 0.08).

### CBI acceptability

Six in-depth group interviews with a total of 20 HCPs (14 nurses and 6 LVHWs) and 28 caregivers at five CBI sites were conducted between February 2017 and June 2018. The mean age among HCPs was 38±12 years, 95% were female, and 35% had at least 7 years of TB-related experience. Among caregivers, mean age was 41±17 years, 93% were female, 21% completed high school, and 7% had a college degree.

#### Identification of child contacts

At CBI sites VHWs visited households of TB patients to ensure that all household contacts were investigated. In one CBI site VHWs were not active during most of the study period as they were striking due to not getting paid; nurses therefore had to rely exclusively on passive invitation.

“We make those patients aware after finding their history that those who have children who are <5 years of age need to be included in the IPT program, the importance of those children to come to the center… So we ask patients, it was supposed to be the VHWs who go to the villages but our area had a problem of VHWs who refuse to follow-up those children in the village because they said they have not been paid.” (Female nurse #1, site 4)

Caregivers sometimes expressed anger at TB patients for exposing their children to TB.

“When she arrived and told me [that my child was exposed to TB] I was not happy, and I said, ‘I have left my place and came to help you like my sister, and look how my child is at risk at the end…’ I was not happy.” (Female caregiver #2, site 4)

#### Home screening

Nurses noted that it is important to send VHWs to follow-up on identification and screening in the home because TB patients do not always remember or are reluctant to name all child contacts and VHWs can screen those children who do not present at the HF.

“…when you ask the patient, maybe they are overwhelmed by the sickness… they don’t provide all the names of people they are living with–… So this one [form] given to the VHWs that the workers should fill it at home and screen those people who do not come to the center, I think it makes a great difference.” (Female nurse #1, site 9)

VHWs conducted TB symptom screening of the children at home, though some caregivers did not realize their children were being screened for TB and thought the VHW just said to bring children to the HF.

”She [VHW] asked me whether the children are eating well and I said they are eating well, and she asked whether they are coughing, and I said no they don’t cough.” (Female caregiver #5, site 6)

“I was called to the health facility [by the LVHW] and told that I should come with the children. The VHW also came to my home and asked to bring the children to the health facility.” (Female caregiver #4, site 3)

VHWs and LVHWs also provided some health education in the home, which helped motivate caregivers to bring their children for screening.

“…they did come, the VHWs… immediately after it was discovered that we live with TB patient in the house, the explanation was so clear that I immediately had to go and fetch the family members because I could not even understand how this one got infected with TB, where, when and how… That gave me an understanding that we were all at risk of being infected, so we all came.” (Female caregiver #5, site 1)

In some cases when the family was not home for the VHW visit, a message was relayed to caregivers to bring children to the HF.

“I was at school at that time [when the VHW came to the house], but I was informed that such people did arrive, informing them that people who live with TB patient must go to the health facility to check, just in case the entire family is infected.” (Male caregiver #5, site 3)

#### Screening at the health facility

Most caregivers did not find TB screening at the HF burdensome because they had been informed about its importance. Some felt it was dangerous not to screen for TB.

“I like it because it helps us to feel free in the house in the presence of TB patient to an extent that I immediately managed to bring all the family members here… I understood its importance when it was explained to me.” (Female caregiver #5, site 1)

“Can you really imagine the situation where a small child suffers from TB? I am worried about such a situation!!” (Male caregiver #6, site 3)

Caregivers reported that the benefit of providing TB services, including TB screening and TPT, is that TB is prevented in the child whereas HCPs saw the benefit as two-fold, protecting both the individual child and the community.

“I understand its [screening] importance because it is for the benefit of the future of the child, so that the child cannot blame me, and that the child doesn’t get TB… it is for her own life.” (Female caregiver #2, site 4)

“… they [child contacts] should not have TB and the community people should be protected.” (Female nurse #1, site 1)

HCPs were frustrated by caregivers who believed that if the TB exposure was limited, then TB screening was not warranted.

“At times the patient will say that the child doesn’t have TB as her husband is working at the mines and he normally comes after a week. And I always say ‘no, to be infected doesn’t mean he comes after a certain period; the fact is children… have been exposed whether for short period or not’ (Female LVHW #1, site 11)

#### Child removal from TB household

Sometimes caregivers were angered about the TB exposure, especially if they themselves did not have TB. As a result, some caregivers removed the child from the household before TB screening was conducted.

“This grandmother said, ‘I am going to bring the child’, at some point for consent sake she told the mother and the father of the child that, ‘I was diagnosed with TB so I am going to take the child to the clinic, it was said the child needs protection.’ The mother then took the child and they [mother and child] were gone.” (Female nurse #1, site 4)

#### Stigma

Caregivers did not report that they were stigmatized because of screening at home or at the health facility.

“It does not bring stigma mostly because it prevents TB so yah I didn’t mind at all.” (Female caregiver #1, site 4)

“This issue is important, mostly because it is not pleasant for a young child to contract TB, and yet the services are there in the hospital. I find this issue to be important and no shame at all in attending such services” (Female caregiver #1, site 1)

## Discussion

Despite a decade-long recommendation by WHO to conduct CCM in high TB burden countries, most child TB contacts in these settings are neither identified, nor screened and evaluated for TB, and therefore do not receive appropriate management for treatment or prevention of TB [7]. Identifying at-risk children is a critical step in the CCM cascade; however, it must be followed by screening them for TB and managing them appropriately thereafter. This study integrated quantitative and qualitative methods, capitalizing on each method’s strengths, to garner important contextual data alongside quantitative results and assist in interpreting study findings. We evaluated a combination CBI for CCM and found that while CBI was acceptable to caregivers and HCPs, the yield of child contacts identified and screened for TB was marginally higher at CBI compared with SOC sites.

Despite historically poor record keeping reported in low resource settings [7], we were able to locate a high percentage of TB cards (95%). At CBI sites where strong mentoring on the importance of CCM as well as monthly multidisciplinary meetings to discuss data were integral components of the intervention, our data showed documentation of child contacts was better, with almost all TB cards having a completed contact section. However, at SOC sites where usual care was provided, less than two-thirds of TB cards had a completed contact section. This difference demonstrates a missed opportunity for identifying and screening TB-exposed children.

Educating TB patients about TB transmission and asking about their child contacts at the HF is crucial [7]. It is also important to conduct follow-up at TB patients’ homes as they may not name all child contacts. The home visit also provides an opportunity to screen child contacts and inform all household members about TB transmission. Health education provided at the home may motivate caregivers to bring their children for further evaluation at the HF and eventually TPT provision, if eligible.

Both HCPs and caregivers in our study reported extensive child movement between households, which made finding and screening children challenging. Caregivers sometimes removed the child from the household where TB contact occurred not realizing that the exposure had already taken place, and that removal of the child from the home did not mitigate the possible need for TB treatment or TPT. This represents an important missed opportunity which may be remedied with further education about TB exposure and risks.

The fertility rate in Lesotho at the time of the study was 3.3 children [21], and therefore we expected a higher number of child contacts identified per adult TB patient. However, the number of child contacts identified was low overall in our study, with 4 children per 10 adults identified. This is in contrast to findings from a South African study, where 7 child contacts per 10 adult TB case were identified [22]. It has been shown that many at-risk children are not identified and documented due to misperceptions regarding the meaning of household composition and children’s TB exposure risk, e.g., duration of exposure, proximity and infectiousness of the adult source patient [23]. Most programs use passive contact tracing—i.e., asking adult TB patients to bring their children to the HF for assessment. HCPs in most high TB burden countries lack tools or guidance on how to define a household and assess TB exposure risk. This leads to confusion on who constitutes a child contact. Furthermore, children may be exposed to and spend substantial amounts of time with adults with TB who do not live in their household (e.g., caregivers). Thus, there is a need for broadening the definition of household as well as a diligent and detailed risk assessment beyond household exposure.

Pervasive stigma was reported as a barrier to identifying and screening TB-exposed children in prior studies [7]. In our study, caregivers in CBI sites did not report stigma associated with screening in the home or HF, which may be due to selection bias as interviews were only conducted with caregivers who brought their children for screening despite attempts to speak with caregivers of children who were not screened. Lack of reported stigma may also be attributable to extensive education provided both in the community and HFs. Studies from high TB/HIV burden countries indicate that caregivers believed child contact investigation could lead to unwanted disclosure of TB and/or HIV status of adult TB patients to family members, friends, and neighbors [7, 23–27]. Fear of stigma can prevent adult TB patients from informing the caregiver of a child contact about the exposure, and thus failing to report the contact to HCPs. Even if a child’s caregiver is made aware, they may not seek follow-up due to stigma. Continued sensitization of HCPs and communities through broad-based health education/TB campaigns would likely increase knowledge, reduce stigma, and change attitudes and perceptions regarding CCM so at-risk children will be identified, screened and evaluated for TB in a timely manner.

Our study had some limitations. As the study was conducted in a real-world setting, we relied on routinely collected programmatic data for our study outcomes, which often were incomplete. However, we were able to locate 95% of TB cards. In addition, we observed a Hawthorne effect, with SOC sites dramatically improving their record-keeping over the study period in conjunction with the HCPs’ observation of study staff collecting CCM data. Furthermore, the number of child contacts identified was lower than assumed in sample size calculations, which limited our power to detect differences between study arms. Nevertheless, we detected a marginally significant and programmatically meaningful difference in yield of TB child contacts between study arms. We did not conduct qualitative interviews at SOC sites; however, as we were assessing CBI acceptability it wasn’t feasible.

In summary, in this mixed-methods cluster-randomized implementation science study, we found that delivery of a multifaceted CBI improved the yield of child TB contacts, a population at risk for developing TB, with potential severe sequalae. Further standardization of child contact identification, including a clear and more inclusive definition of household contacts, expansion of education regarding TB exposure and associated risks and stigma-reduction interventions are required to increase the yield of CCM and prevent TB in this vulnerable population.

## Supporting information

S1 Checklist (DOCX)

S1 File (PDF)
